# Neuronal-Specific Inhibition of Endoplasmic Reticulum Mg^2+^/Ca^2+^ ATPase Ca^2+^ Uptake in a Mixed Primary Hippocampal Culture Model of Status Epilepticus

**DOI:** 10.3390/brainsci10070438

**Published:** 2020-07-10

**Authors:** Laxmikant S. Deshpande, Robert J. DeLorenzo, Severn B. Churn, J. Travis Parsons

**Affiliations:** 1 Department of Neurology, Virginia Commonwealth University, Richmond, VA 23298, USA; laxmikant.deshpande@vcuhealth.org (L.S.D.); robert.delorenzo@vcuhealth.org (R.J.D.); severn.churn@nih.gov (S.B.C.); 2 Department of Pharmacology and Toxicology, Virginia Commonwealth University, Richmond, VA 23298, USA; 3 Department of Biochemistry and Molecular Biophysics, Virginia Commonwealth University, Richmond, VA 23298, USA; 4 Department of Physiology, Virginia Commonwealth University, Richmond, VA 23298, USA; 5 Department of Anatomy and Neurobiology, Virginia Commonwealth University, Richmond, VA 23298, USA

**Keywords:** seizure, epilepsy, SERCA, NMDA, glia, calcium homeostasis

## Abstract

Loss of intracellular calcium homeostasis is an established mechanism associated with neuronal dysfunction and status epilepticus. Sequestration of free cytosolic calcium into endoplasmic reticulum by Mg^2+^/Ca^2+^ adenosinetriphosphatase (ATPase) is critical for maintenance of intracellular calcium homeostasis. Exposing hippocampal cultures to low-magnesium media is a well-accepted in vitro model of status epilepticus. Using this model, it was shown that endoplasmic reticulum Ca^2+^ uptake was significantly inhibited in homogenates from cultures demonstrating electrophysiological seizure phenotypes. Calcium uptake was mainly neuronal. However, glial Ca^2+^ uptake was also significantly inhibited. Viability of neurons exposed to low magnesium was similar to neurons exposed to control solutions. Finally, it was demonstrated that Ca^2+^ uptake inhibition and intracellular free Ca^2+^ levels increased in parallel with increasing incubation in low magnesium. The results suggest that inhibition of Mg^2+^/Ca^2+^ ATPase-mediated endoplasmic reticulum Ca^2+^ sequestration contributes to loss of intracellular Ca^2+^ homeostasis associated with status epilepticus. This study describes for the first time inhibition of endoplasmic reticulum Mg^2+^/Ca^2+^ ATPase in a mixed primary hippocampal model of status epilepticus. In combination with animal models of status epilepticus, the cell culture model provides a powerful tool to further elucidate mechanisms that result in inhibition of Mg^2+^/Ca^2+^ ATPase and downstream consequences of decreased enzyme activity.

## 1. Introduction

Status epilepticus (SE) is a deleterious neurological seizure disorder attributed to a considerable rate of morbidity and mortality in children and adults [1,2]. Many regions of the brain are afflicted by SE, but the hippocampus is of particular interest due to its high sensitivity to trauma and its central role in learning and memory [3,4,5]. Associated with cellular damage and dysfunction in the hippocampus as a result of prolonged seizure activity is loss of intracellular free Ca^2+^ ([Ca^2+^]_i_) homeostasis. This has been demonstrated in both animal [6,7,8] and cell culture [9,10,11] models of SE. While animal models are important for an in vivo understanding of prolonged seizure disorders and have a closer resemblance to human pathology, neuronal cell cultures provide an important in vitro tool for the elucidation of intercellular and intracellular molecular mechanisms associated with SE.

Exposure of primary hippocampal cultures to low Mg^2+^ media is an accepted and well characterized model of SE in the culture dish that has been routinely used to carry out electrophysiological, biochemical, and molecular investigations [10,11,12,13,14,15]. Whole-cell patch-clamp studies showed that neurons displayed continuous tonic high-frequency epileptiform discharges, similar to electroencephalographic patterns seen in animal models [16,17], throughout the standard 3 h incubation of the cultures in low Mg^2+^ media. Several lines of evidence revealed altered [Ca^2+^]_i_ physiology and cellular dysfunction in hippocampal neurons examined acutely after exposure to 3 h of low Mg^2+^ solution. It was demonstrated that functioning of key Ca^2+^-mediated enzymes, such as Ca^2+^/calmodulin-dependent protein kinase II [18], and Ca^2+^-dependent trafficking of critical post-synaptic inhibitory neurotransmitter receptors, such as the gamma-aminobutyric acid type A receptor [13], were altered. Most importantly, it was revealed that [Ca^2+^]_i_ levels were significantly elevated throughout and acutely after 3 h of exposure of hippocampal cultures to low Mg^2+^ media [15]. This prolonged elevation of [Ca^2+^]_i_ would have profound consequences for Ca^2+^-dependent enzymes and protein trafficking.

One important mechanism for the proper maintenance of [Ca^2+^]_i_ homeostasis in neurons is sequestration of Ca^2+^ from the cytosol to the lumen of the endoplasmic reticulum (ER) mediated by the ER membrane-bound sarco/endoplasmic reticulum Mg^2+^/Ca^2+^ ATPase (SERCA). SERCA is a member of the p-type ATPase family which requires adenosine triphosphate (ATP) to transport Ca^2+^ from the cytoplasm to the ER lumen against a concentration gradient. Briefly, Mg^2+^-ATP binds to SERCA forming a high-energy phosphorylated intermediate by reaction of the gamma phosphate of ATP to an aspartate residue of the catalytic domain [19]. The energy garnered is used to drive 2 Ca^2+^ molecules into the ER from the cytoplasm for every ATP molecule hydrolyzed into adenosine diphosphate + phosphate. Our lab showed in several models of neurological disorders associated with loss of [Ca^2+^]_i_ homeostasis that SERCA-mediated Ca^2+^ uptake into microsomes (ER vesicles) isolated from brain homogenates was significantly inhibited [20,21,22]. In cortex, we demonstrated that SERCA-mediated Ca^2+^ uptake was significantly decreased in microsomes isolated acutely after 1 h of pilocarpine-induced SE in the rat [21]. In the same model, we revealed that inhibition of SERCA-mediated Ca^2+^ uptake in cortex microsomes was dependent upon N-methyl-D-aspartate (NMDA) receptor activation [21] and that this inhibition persisted for one year into the chronic epilepsy phase [20]. We also developed a protocol that directly measures neuronal-specific SERCA-mediated Ca^2+^ uptake in homogenates of primary hippocampal cultures [23]. In the present manuscript, this method was employed to characterize inhibition of neuronal-specific SERCA-mediated Ca^2+^ uptake in homogenates acutely isolated from primary hippocampal cultures subjected to low Mg^2+^-induced SE. Presented for the first time in this manuscript is inhibition of neuronal and glial SERCA-mediated Ca^2+^ uptake prepared from hippocampal cell cultures associated with exposure to low Mg^2+^ for 3 h. This in vitro SE model, when paired with established in vivo animal models, will help to elucidate both upstream mechanisms that cause inhibition of SERCA and downstream aftermaths associated with Mg^2+^/Ca^2+^ ATPase activity.

## 2. Materials and Methods

### 2.1. Materials

All materials used were the same as described in Parsons et al. [23] and purchased from Fisher Scientific (Pittsburgh, PA, USA), Sigma Chemical Co. (St. Louis, MO, USA), or Bio-Rad (Richmond, CA, USA) except for the cell culture materials described in Sun et al. [24] and other materials described below.

### 2.2. Primary Hippocampal Cell Cultures

All animal use procedures were in strict accordance with the National Institutes of Health Guide for the Care and Use of Laboratory Animals and approved by Virginia Commonwealth University’s Institutional Animal Care and Use Committee (protocol AM10147). Primary mixed neuronal cultures were prepared as previously described [23,24] with the following modifications. Cells were plated on polystyrene dishes (35 mm diameter) after an overnight pretreatment with 0.05 mg/mL poly-l-lysine followed by multiple rinses with distilled H_2_O. Glia were plated at a density of 5.0 × 10^4^ per dish and grown for two weeks to reach confluence. Neurons were then plated on the confluent glial beds at a density of 2.7 × 10^5^ per dish unless otherwise stated. Once neurons were plated, cultures were maintained for 2 weeks in vitro prior to use for experimental manipulation. When glial cultures were grown for experimental comparison to neurons grown on confluent glial beds (N+G), the glia were grown in parallel and treated in an identical fashion to N+G cultures with the exception that neurons were not plated. In both cases, each culture dish represents 1 n value.

### 2.3. SE in the Culture Dish

SE was induced in primary hippocampal cell cultures as described previously [12]. Briefly, dishes were removed from the incubator and washed with 2 × 1 mL with physiological recording (control) solution (145 mM NaCl, 2.5 mM KCl, 2.0 mM CaCl_2_, 10 mM glucose, 2.0 μM glycine, 1.0 mM MgCl_2_, 10 mM 4-(2-hydroxyethyl) piperazine-1-ethanesulfonic acid (HEPES), pH 7.2, and osmolarity adjusted to 325 ± 10 mOsm with sucrose) or low Mg^2+^ solution (the same as control solution without added MgCl_2_). The cells were then incubated in 1 mL of either control or low Mg^2+^ solution for 1 to 3 h at 37 °C in 5.0% CO_2_/95% air. Immediately proceeding the incubation, dishes were removed from the incubator, put on ice, and washed 3 × 1 mL with homogenization buffer (320 mM sucrose, 1 mM EGTA, 1 mM EDTA, 1 mM dithiothreitol, 0.3 mM phenylmethylsulfonyl fluoride, and 5 mM HEPES, pH 6.9–7.0). Cells were then scraped in 150 μL of homogenization buffer per dish, homogenized with Duall 20 handheld glass homogenizers (Kontes Glass, Vineland, NJ, USA), and stored at −80 °C until used. For cell viability studies, dishes were blindly observed by an unbiased investigator immediately following 3 h of incubation and just prior to homogenization using the criteria of phase brightness [18,25,26]. Neurons were classified as either viable (phase bright) or non-viable (phase dark) using an Olympus CK2 inverted microscope (Olympus America, Melville, NY, USA) outfitted with 20× objective and phase filters. Phase images were captured with Q Capture (version 2.55, Quantitative Imaging, Burnaby, British Columbia, Canada) using an Olympus Q Fire digital camera mounted on an Olympus CK-40 microscope equipped with phase filters. Because neurons in culture do not contain true physiological anatomical connections or clinical seizures, data must be carefully interpreted as an in vitro model that provides insight to the molecular mechanisms underlying [Ca^2+^]_i_ dysfunction due to prolonged depolarization/repolarization events.

### 2.4. Electrophysiological Monitoring of SE in Hippocampal Neurons

Whole-cell current-clamp electrophysiological studies were performed as previously described [12,13,27]. Briefly, cultures were incubated in control or low Mg^2+^ solutions as described above. Cultures were then transferred to the stage of an Olympus IX-70 inverted microscope equipped with phase contrast optics and were perfused continuously (gravity perfusion) at 1 mL/min with low Mg^2+^ or control solutions throughout each experiment. Temperature was maintained at 34–35 °C using a heating stage (Brook Industries, Lake Villa, IL, USA). Patch electrodes with a resistance of 2–5 MΩ were pulled from borosilicate glass capillaries (WPI, Sarasota, FL, USA) using a Brown-Flaming PC-80 puller (Sutter Instruments, Novato, CA, USA) and then fire polished. Electrodes were filled with a solution containing 140 mM potassium gluconate, 1 mM MgCl_2_, 10 mM sodium HEPES, pH 7.2, and osmolarity adjusted to 310 ± 5 mOsm with sucrose. Recordings were conducted in the whole-cell current-clamp mode [28] using an Axopatch 200A amplifier (Axon Instruments, Foster City, CA, USA). Data were digitized and stored on videotape using a Neurocorder (Neurodata, Cygnus Technology, Delaware Water Gap, PA, USA) and played back for analysis on a Dash IV chart recorder (Astro-Med, Warwick, RI, USA).

### 2.5. Determination of [Ca^2+^]_i_ Levels in Hippocampal Culture Neurons Using Fluorescent Ca^2+^ Imaging

Changes in neuronal [Ca^2+^]_i_ were measured using the ratiometric, high-affinity fluorescent calcium indicator, fura-2 acetoxymethyl ester (fura-2 AM, Molecular Probes, Eugene, OR, USA), employing modified procedures previously established [29,30]. For imaging experiments, cells (plated at a density of 1.0 × 10^5^ neurons on 5.0 × 10^4^ glia) were grown on Lab-Tek coverglass chambers (Nalge Nunc International, Rochester, NY, USA) as described above. Neurons plated on glia were incubated in control or low Mg^2+^ solution for either 20 min (early) or 2 h and 20 min (late) followed by incubation in control or low Mg^2+^ solution plus 1 µM fura-2 AM (0.1% *v/v* DMSO) for 30 min. Cells were then washed 3 times in respective control or low Mg^2+^ solution and incubated for an additional 10 min in control or low Mg^2+^ solution to allow for complete cleavage of fura-2 AM. All incubation procedures were at 37 °C in a 5% CO_2_/95% air atmosphere. Chambers were then transferred to a heated 37 °C stage (Harvard Apparatus, Holliston, MA, USA) of an Olympus IX 70 inverted microscope and cells were visualized using a 20 × 0.7 numerical aperture fluorite water-immersion objective. Fura-2 AM was excited with a 75 W xenon arc lamp (Olympus Optical, Shinjuku-ku, Tokyo, Japan) and ratio images were acquired by alternating excitation wavelengths (340/380) by using a Lambda 10-2 filter wheel (Sutter Instruments Co., Novato, CA, USA) and a filter cube at 510/540 emission with a dichroic at 400 nm using a cooled digital CCD camera (LSR AstroCam Limited, Cambridge, England). Only neurons displaying normal morphology (healthy somata, visible nucleus, intact processes) were picked for analysis. The cells were chosen prior to fluorescence and following control or low Mg^2+^ incubation using the Olympus IX 70 and phase contrast filters. All imaging data and figures were generated using the temporal module of Ultraview Imaging Suite 5.2 (Perkin Elmer, Wellesley, MA, USA).

### 2.6. ER-Mediated Ca^2+^ Uptake Measured in the Unfractionated Homogenates

Measurement of ^45^Ca^2+^ transport from external media into ER vesicles mediated by SERCA was measured as previously described [21] and modified by Parsons et al. [23]. Final homogenate concentration in the reactions was 0.18 μg/μL unless otherwise specified. Homogenate protein concentrations were determined by the method of Bradford [31]. Radioactive calcium stock was prepared by diluting 1 mCi ^45^CaCl_2_ 1:10 with a 100 mM ^40^CaCl_2_ standard which was then adjusted to 6 mM. The 6 mM stock solution had a radioactivity of 151 µCi/µmol of total ^45^CaCl_2_:^40^CaCl_2_. Therefore, the measurement of both ^40^Ca^2+^- and ^45^Ca^2+^-mediated Ca^2+^ uptake by SERCA could be determined by the ^45^Ca^2+^ tracer. Briefly, all components of the Ca^2+^ uptake assay (homogenate, 1 mM EGTA assay buffer, 100 mM KCl, 10 mM oxalate, and 1 mM MgCl_2_, all final concentrations) were added to test tubes on ice. Test tubes were then placed in a shaking water bath set to 37 °C and ATP (2 mM final) was added immediately. Once at 37 °C, 600 μM radioactive CaCl_2_ (final concentration) was added to start the reaction. At specified time points, the reaction was terminated by vacuum filtration and Ca^2+^ uptake quantified using liquid scintillation spectrometry.

### 2.7. Data and Statistical Analyses

Numerical, graphical, and statistical analyses were performed using Prism 6 (GraphPad Software, San Diego, CA, USA). All data presented are the mean of replicate determinations ± standard error of the mean.

## 3. Results

### 3.1. Electrophysiological Monitoring of SE in Hippocampal Neurons

**Figure 1:**
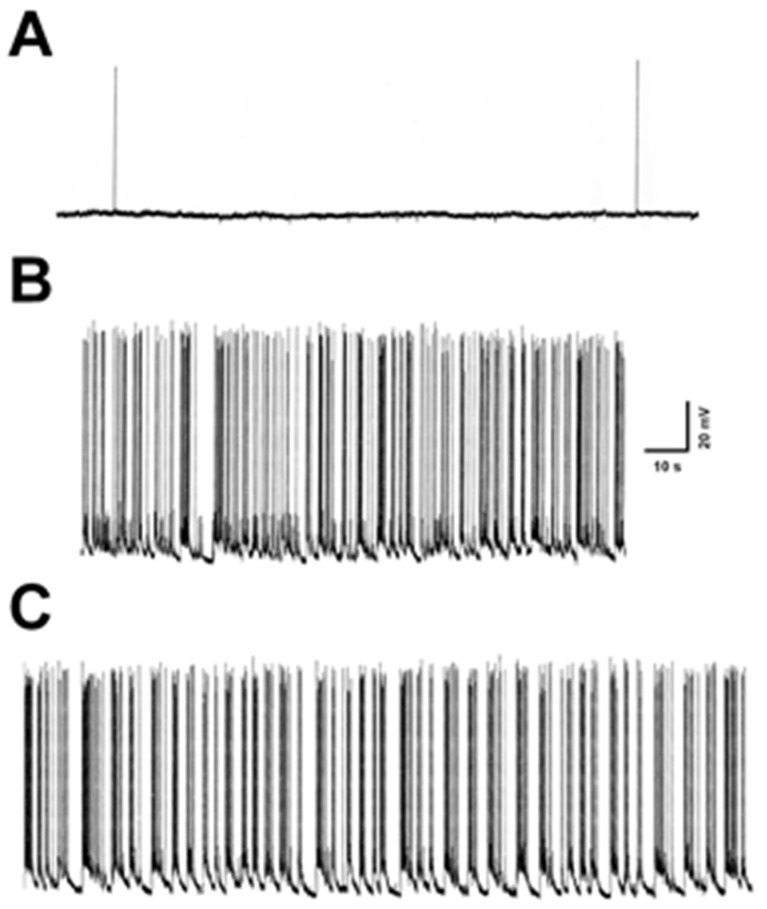
Electrophysiological monitoring of SE in hippocampal neurons. Culture dishes were incubated in control or low Mg^2+^ solutions and neurons were patch clamped using the whole-cell current-clamp mode as described in Materials and Methods. Scale bar described in part B is applicable to all traces. (**A**) Representative recording of a neuron incubated in control solution. (**B**) Representative trace of a neuron incubated in low Mg^2+^ solution for approximately 1 h. (**C**) Representative recording of a neuron incubated in low Mg^2+^ solution for approximately 3 h.

In order to determine the effect of low Mg^2+^ exposure on SERCA-mediated Ca^2+^ uptake in hippocampal culture homogenates, it was important to confirm continuous spike discharges in a network of cells incubated in low Mg^2+^ media. Spike discharges in hippocampal neurons in culture are commonly monitored using whole-cell current-clamp methodology [27,32,33]. Figure 1 shows typical patch-clamp traces of neurons incubated in control or low Mg^2+^ solutions. Control neurons exhibited a mean membrane potential of −61.2 ± 2.5 mV and a mean input resistance of 120.6 ± 7.4 MΩ, whereas the low Mg^2+^ neurons demonstrated a mean membrane potential of −59.8 ± 2.1 mV and a mean input resistance of 118.9 ± 8.4 MΩ. No significant differences in resting membrane potential or input resistance were observed between control and low Mg^2+^ (*n* = 6, *p* > 0.05, *t*-test). The spike discharges following removal of Mg^2+^ from the incubating medium were greater than 3 Hz (5–15 Hz) and meet the electrographic definition of SE. Three to 5 neurons/plate were routinely patched to confirm presence of spike discharges and all the successfully patched neurons showed high-frequency spiking. It was previously shown that this activity is not limited to few cells but is a network phenomenon and occurs synchronously in populations of neurons [12]. These data show that neurons acutely harvested as N+G cultures after incubation for 3 h in low Mg^2+^ solution displayed spike discharge activity for the duration of the treatment protocol.

### 3.2. Inhibition of SERCA-Mediated Ca^2+^ Uptake in N+G Homogenates Exposed to Low Mg^2+^ Media

**Figure 2:**
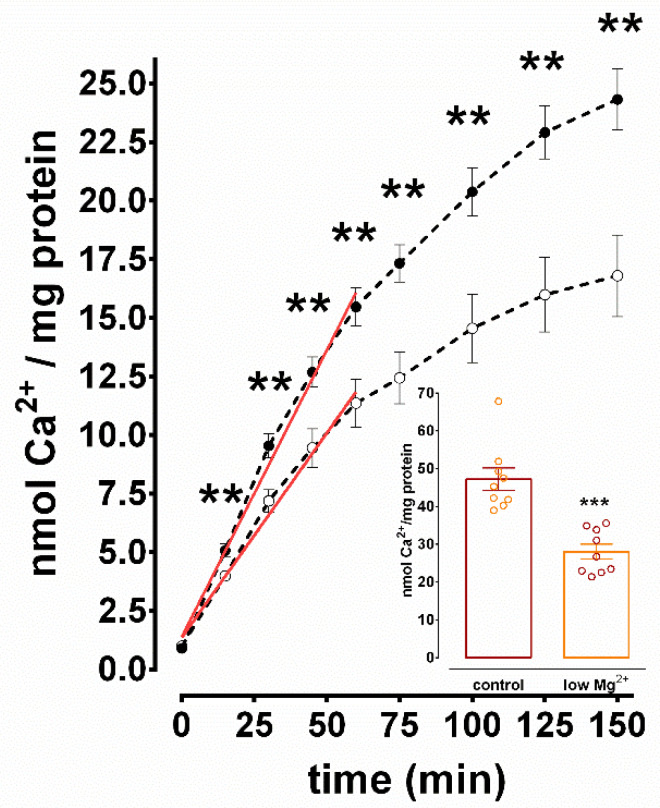
Inhibition of Ca^2+^ uptake in mixed hippocampal culture homogenates exposed to low Mg^2+^ media for 3 h. Homogenates were isolated acutely from N+G cultures incubated in either control (closed circles) or low Mg^2+^ solution (open circles) for 3 h and standard Ca^2+^ uptake reactions were performed as described under Materials and Methods. Two-tailed unpaired *t*-test was used to compare each time point of Ca^2+^ uptake in control to low Mg^2+^ homogenates (** *p* ≤ 0.008, all time points ≥ 15 min, *n* = 10 for all). Red lines represent linear regression slopes of the data points from 5 s to 60 min. Figure 2 (inset): Inhibition of Ca^2+^ uptake in mixed hippocampal culture homogenates from neurons subjected to electrophysiological monitoring during treatment. Activity was determined after 150 min of Ca^2+^ uptake following 3 h of incubation in either control or low Mg^2+^ solution. *** *p* < 0.0001, *n* = 9 each, two-tailed unpaired *t*-test.

It was previously demonstrated that Ca^2+^ uptake measured in N+G homogenates was specifically ER Mg^2+^/Ca^2+^ ATPase mediated [23]. Total Ca^2+^ uptake was measured in homogenates isolated acutely from N+G cultures incubated for 3 h in either control or low Mg^2+^ solutions (Figure 2). SE resulted in significant inhibition of Ca^2+^ uptake compared to control at all time points ≥ 15 min. Over all of the time points measured, Figure 2 reveals that low Mg^2+^ caused a maximal inhibition of 30.9 ± 7.2% at the 150 min time point. In several experiments, N+G homogenates of hippocampal cultures subjected to electrophysiological analysis were prepared and tested for Ca^2+^ uptake in parallel (Figure 2 inset). After 150 min of Ca^2+^ uptake, N+G homogenates that underwent 3 h of SE and neurons that displayed prolonged and continuous electrical discharges had a significant 40.6 ± 4.1% inhibition of Ca^2+^ sequestration compared to controls.

Since increasing Ca^2+^ accumulation within ER vesicles over time results in product inhibition of the SERCA enzyme, it was important to investigate the effect of SE on the initial rate of Ca^2+^ uptake. The data points from 5 s to 60 min in Figure 2 were subjected to linear regression analysis (r^2^ = 0.99). SE resulted in a significant 28.7 ± 7.2% inhibition (0.25 ± 0.01 vs. 0.17 ± 0.01 nmol Ca^2+^/mg protein/min, control vs. low Mg^2+^, respectively, two-tailed unpaired *t*-test, *p* = 0.005, *n* = 10) of the initial rates of Ca^2+^ sequestration calculated from the slopes of the regression lines compared to control. The data from Figure 2 demonstrate that 3 h of continuous spike discharge activity in hippocampal culture dish result in significant inhibition of SERCA activity when measured in N+G homogenates isolated acutely after the SE event.

**Figure 3:**
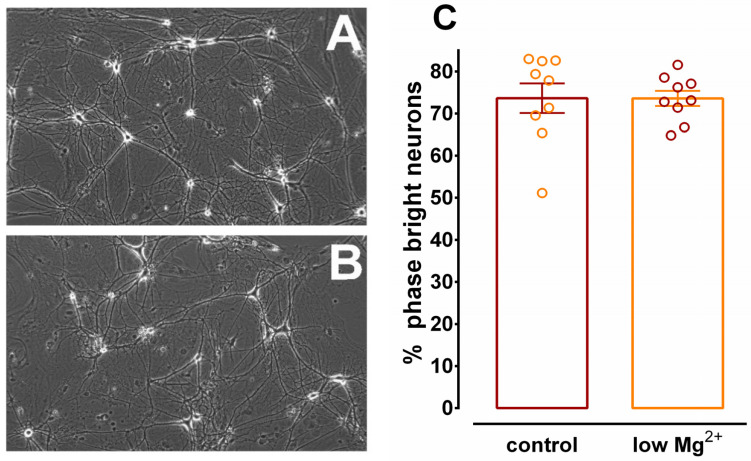
Viability of hippocampal neurons after 3 h of SE. Culture dishes were incubated in control or low Mg^2+^ solutions and assessed for viability by a blind observer as described in Materials and Methods. Representative phase contrast images of cultures exposed for 3 h to either control (**A**) or low Mg^2+^ (**B**) solutions. Images are presented at a 20× magnification. Quantification of neurons (**C**) using the criteria of phase bright (viable) and phase dark (non-viable). The number of phase-bright cells was divided by the total number of cells (phase bright + dark). Two-tailed unpaired *t*-test revealed that there was no significant difference in neuronal viability between the two treatment groups (*p* = 0.99, *n* = 9 dishes each with multiple fields analyzed per dish).

Because hippocampal neurons are extremely sensitive to traumatic insults, it was important to investigate the effect of low Mg^2+^ treatment on cell viability. N+G cultures were incubated in either control or low Mg^2+^ solutions for 3 h. At the end of the incubation period and prior to homogenization for Ca^2+^ uptake assays, the neurons were blindly observed by an unbiased investigator using the criteria of phase brightness [18,25,26]. Both phase-bright and phase-dark neurons were counted. The number of phase-bright neurons were then divided by the total number of neurons counted to yield the percentage of viable neurons. Figure 3A,B depict representative phase images of cultures subjected to 3 h of control or low Mg^2+^ solution, respectively. No noticeable gross anatomical differences in somatic size, dendritic length, or neuronal density were observed. Quantitative analysis of phase-bright and phase-dark neurons is demonstrated in Figure 3C. It was found that there were 73.6 ± 3.5% and 73.6 ± 1.8% viable neurons in control and low Mg^2+^ cultures, respectively. The data from Figure 3 demonstrate that the inhibition of Ca^2+^ uptake in N+G homogenates observed in Figure 2 was not due to significant cell loss in the cultures subjected to the low Mg^2+^ protocol.

### 3.3. Inhibition of Ca^2+^ Uptake in Homogenates of Glia Incubated in Low Mg^2+^

**Figure 4:**
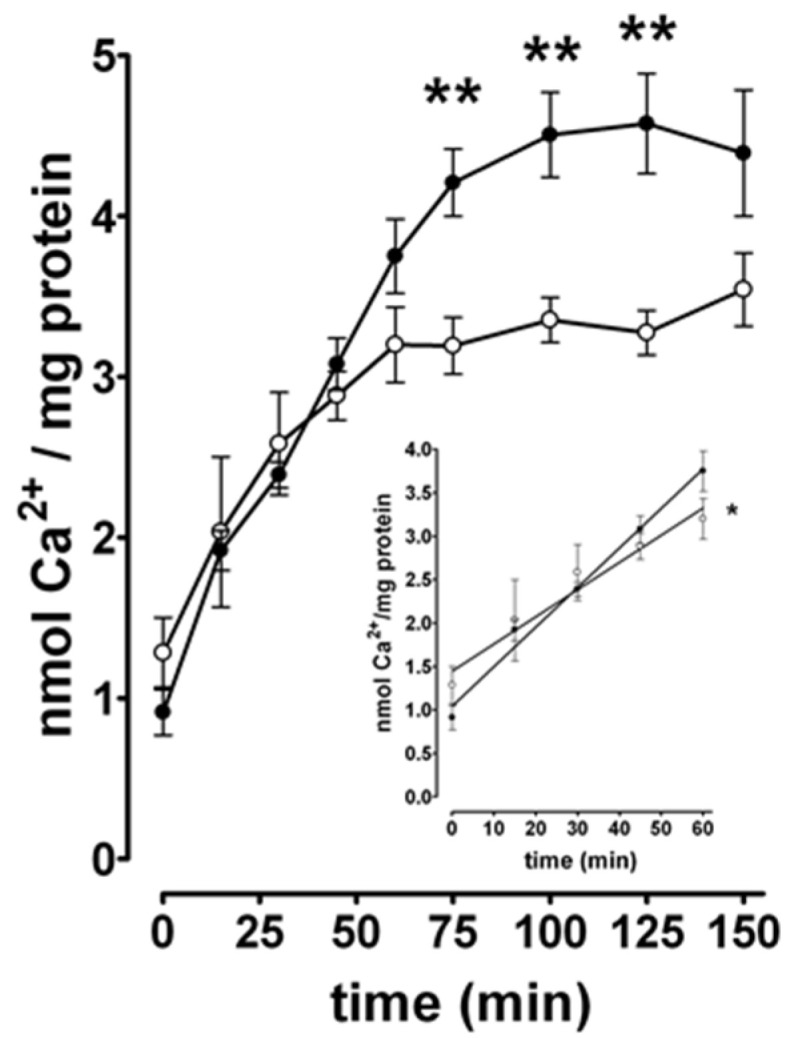
Low Mg^2+^-induced inhibition of total and initial rate of Ca^2+^ uptake in hippocampal glial culture homogenates. Homogenates were isolated acutely from glial cultures incubated in either control (closed circles) or low Mg^2+^ solution (open circles) for 3 h and standard Ca^2+^ uptake reactions were performed as described under Materials and Methods. Two-tailed unpaired *t*-test was used to compare each time point of Ca^2+^ uptake in control to low Mg^2+^ homogenates (** *p* ≤ 0.004, 75, 100, and 125 min time points, *n* = 6 for both). Figure 4 (inset): Data points from 5 s to 60 min were subjected to linear regression analysis. Initial rates were determined from the slopes of the linear regression lines. Two-tailed unpaired *t*-test revealed that the initial rate of Ca^2+^ uptake in low Mg^2+^ glial homogenates is significantly less than the initial rate of Ca^2+^ uptake in control glial homogenates (* *p* = 0.01, *n* = 6 for both).

Primary hippocampal neurons utilized in this study were grown on a confluent bed of glial cells. Since N+G homogenates used for Ca^2+^ uptake studies consisted of glial cells in addition to neuronal cells, it was important to test the effect of low Mg^2+^ treatment on the Ca^2+^ sequestration capacity of glial homogenates. Total Ca^2+^ uptake was measured in homogenates isolated acutely from glial cultures incubated in either control or low Mg^2+^ solutions for 3 h (Figure 4). There was significant inhibition of Ca^2+^ uptake in glial homogenates subjected to low Mg^2+^ treatment compared to control at the 75, 100, and 125 min time points. Over all of the time points measured, Figure 4 reveals that incubation in low Mg^2+^ media caused a maximal inhibition of 28.4 ± 3.0% at the 125 min time point. Visual observation of the data in Figure 4 suggests that there is a difference in the initial rates of Ca^2+^ uptake in control versus low Mg^2+^ treated glial homogenates. The data from 5 s to 60 min of Ca^2+^ uptake in Figure 4 were subjected to linear regression analysis (Figure 4 inset). The initial rates as determined from the slopes of the linear regression lines were 0.046 ± 0.004 and 0.031 ± 0.003 nmol Ca^2+^/mg protein × min^−^^1^ for control and low Mg^2+^ glial homogenates, respectively—a significant inhibition of 31.3 ± 5.6%. The results of the glia study show that prolonged exposure to low Mg^2+^ solution caused similar inhibition of the initial rates and total Ca^2+^ uptake in glial compared to N+G homogenates.

### 3.4. Inhibition of SERCA-Mediated Ca^2+^ Uptake in Hippocampal N+G Homogenates Is Duration Dependent

**Figure 5:**
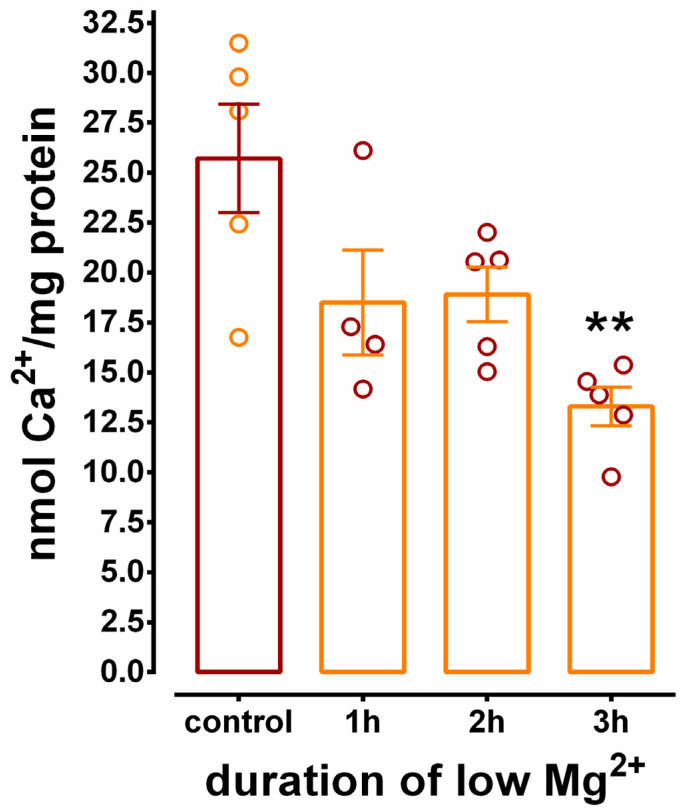
The effect of low Mg^2+^ exposure duration of mixed hippocampal cultures on SERCA activity measured at 150 min of Ca^2+^ uptake. Homogenates were isolated acutely from N+G cultures incubated in either control solution for 3 h or low Mg^2+^ solution for 1, 2, or 3 h and standard Ca^2+^ uptake reactions were performed as described under Materials and Methods. Activity was determined after 150 min of Ca^2+^ uptake. One way ANOVA revealed that incubation in low Mg^2+^ solution significantly inhibited Ca^2+^ uptake (*p* = 0.004, *n* = 5 for all except 1 h, *n* = 4). Tukey post-hoc analysis revealed that 3 h of incubation in low Mg^2+^ was significantly different than 3 h of incubation in control (** *p* < 0.01).

Incubation of hippocampal cultures for 3 h in low Mg^2+^ solution is the established exposure period for the generation of epileptogenesis in this model [11,12,33]. Therefore, it is important to investigate the duration of low Mg^2+^ exposure necessary to cause significant inhibition of SERCA-mediated Ca^2+^ sequestration. Hippocampal N+G cultures were exposed to low Mg^2+^ solution for 1, 2, and 3 h and compared to cultures exposed to control solution for 3 h. Figure 5 examines Ca^2+^ uptake in N+G homogenates acutely isolated from cultures displaying SE for the durations described above. Exposure of hippocampal cultures to low Mg^2+^ solution for 1 h or greater resulted in significant inhibition of Ca^2+^ uptake as measured in N+G homogenates at the 150 min time point of the Ca^2+^ uptake reaction. SERCA-mediated Ca^2+^ sequestration was inhibited 28.1 ± 10.2%, 25.5 ± 5.3%, and 48.3 ± 3.8% at 1, 2, and 3 h, respectively. The data presented in Figure 5 reveal that SERCA-mediated Ca^2+^ uptake is inhibited prior to the exposure period required for low Mg^2+^-induced epileptogenesis.

### 3.5. Changes in [Ca^2+^]_i_ in Hippocampal Neurons During SE

**Figure 6:**
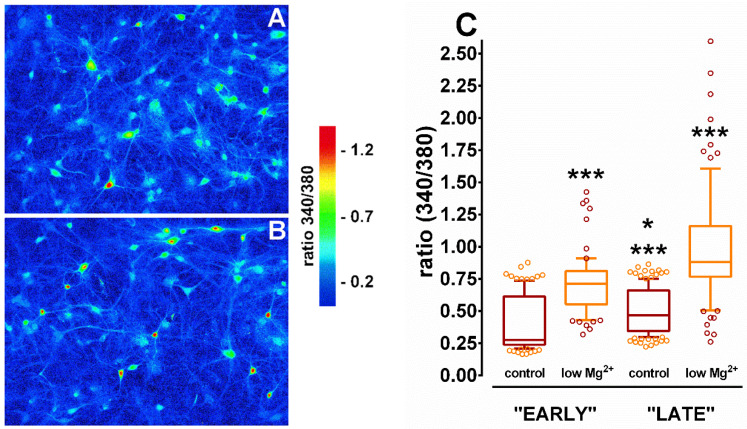
Changes in [Ca^2+^]_i_ in hippocampal neurons during SE. Culture dishes were incubated in control or low Mg^2+^ solutions and [Ca^2+^]_i_ levels were determined using the fluorescent Ca^2+^ binding dye fura-2 AM and standard Ca^2+^ imaging techniques described in Materials and Methods. Representative pseudocolor images of cultures exposed for 3 h to either control (**A**) or low Mg^2+^ (**B**) solutions. Increasing 340/380 ratio values correspond to increasing [Ca^2+^]_i_ concentrations. Images are presented at a 20× magnification. (**C**) Box and whiskers plot (whiskers display 10–90 percentile) quantification of neuronal 340/380 ratio values after 1 h (72 neurons) and 3 h (86 neurons) of low Mg^2+^ incubation compared to 1 h (109 neurons) and 3 h (132 neurons) in control solution from multiple culture dishes. One-way ANOVA (*p* < 0.0001) followed by the Tukey post-hoc test revealed that 3 h low Mg^2+^ vs. 3 h control, 3 h vs. 1 h low Mg^2+^, and 1 h low Mg^2+^ vs. 1 h control were all significantly different (*** *p* < 0.001). Tukey post-hoc test also revealed that 3 h vs. 1 h control was significantly different (* *p* < 0.01).

Since [Ca^2+^]_i_ in both the cytosol and the ER lumen affects SERCA activity, it was important to determine [Ca^2+^]_i_ during SE in hippocampal neurons. N+G cultures were incubated in either control or low Mg^2+^ solution and [Ca^2+^]_i_ levels were determined using a fluorescent [Ca^2+^]_i_ indicator and Ca^2+^ imaging microscopy. Representative pseudocolor images of cultures incubated in control and low Mg^2+^ solutions are shown in Figure 6. It can be seen in the control dish (Figure 6A) that [Ca^2+^]_i_ was slightly elevated in a select population of hippocampal neurons with only 1–2 cells displaying significant increase in [Ca^2+^]_i_. Intracellular free Ca^2+^ was also slightly elevated in a select population of neurons in the low Mg^2+^ dish (Figure 6B). However, a much greater percentage (10–12 cells) displayed significantly elevated [Ca^2+^]_i_. Figure 6C quantitates [Ca^2+^]_i_ concentrations in control and low Mg^2+^ neurons during the early and late phases of treatment as described above. It was revealed that [Ca^2+^]_i_ was significantly increased by 45.9% and 48.2% in low Mg^2+^ compared to control-treated neurons after 1 h and 3 h of incubation, respectively. It was also shown that neurons incubated in control solution for 3 h had a significant 23.7% increase in [Ca^2+^]_i_ compared to neurons incubated in control solution for 1 h. Finally, it was demonstrated that neurons incubated in low Mg^2+^ solution for 3 h had a significant 26.9% increase in [Ca^2+^]_i_ compared to neurons incubated in low Mg^2+^ solution for 1 h. The data reveal that low Mg^2+^ treatment resulted in a significant increase in [Ca^2+^]_i_ during the early phase of incubation that persisted for the duration of the procedure.

## 4. Discussion

Inhibition of SERCA-mediated Ca^2+^ sequestration into ER vesicles as a result of neurological dysfunction has been well documented. It was shown that Ca^2+^ uptake was significantly inhibited in microsomes (ER vesicles) isolated from rat whole brain as a result of global ischemia [22,34]. It was also demonstrated that SERCA-mediated Ca^2+^ uptake was significantly decreased in cortical microsomes isolated acutely after pilocarpine-induced SE in the rat [21]. Furthermore, it was discovered that inhibition of Ca^2+^ uptake in cortex ER vesicles as a result of pilocarpine-induced SE persisted well into the chronic spontaneous recurrent seizure phase in this acquired epilepsy model [20].

Significant inhibition of Ca^2+^ uptake was observed in hippocampal culture homogenates as a result of incubation in low Mg^2+^ solution for 3 h (Figure 2). Several other mechanisms exist in homogenates that sequester Ca^2+^ such as plasma membrane ATPases and mitochondria [35]. However, it was shown using thapsigargin that Ca^2+^ sequestration measured under conditions utilized in this study was ER SERCA specific [23,36]. Multiple scenarios exist that would result in apparent inhibition of ER vesicle Ca^2+^ uptake but not inhibition of SERCA Ca^2+^ transport activity. It is possible that the observed inhibition of Ca^2+^ uptake was due to increased Ca^2+^ efflux from ER vesicles through ryanodine and/or inositol-1,4,5-trisphosphate receptors and/or ER vesicles rendered leaky as a result of low Mg^2+^ treatment. While these mechanisms cannot be ruled out from the current study, the inhibition of the initial rate of Ca^2+^ uptake (Figure 2), before Ca^2+^ release becomes significant, demonstrates specific inhibition of SERCA-mediated Ca^2+^ transport activity.

It is well known that Mg^2+^ is an antagonist of the NMDA receptor ion channel. Thus, upon exposure of neuron cultures to low Mg^2+^, the NMDA ion channel opening is prolonged, allowing excess influx of Ca^2+^ into the cytosol. In turn, Ca^2+^ is sequestered by SERCA into the ER and as lumenal Ca^2+^ increases, SERCA activity will decrease. Therefore, it is possible that the inhibition of SERCA-mediated Ca^2+^ uptake observed in cultures subjected to low Mg^2+^ is the result of more Ca^2+^ accumulated in the ER vesicles compared to control cultures following exposure and prior to assessing SERCA activity. We acknowledge that experiments examining whether ER vesicles are preloaded with Ca^2+^ prior to uptake assays are lacking and cannot be ruled out. However, several lines of evidence suggest that inhibition of SERCA activity is not solely due to increased Ca^2+^ in the microsomes. Oxalate is an anion that creates non-soluble salts with Ca^2+^ in the lumen of ER resulting in a several fold increase in the steady-state capacity of the ER vesicles to sequester Ca^2+^ [37]. Since the reaction medium of the SERCA-mediated Ca^2+^ uptake assay contained 10 mM oxalate, it is likely that the anion precipitated any Ca^2+^ in the ER vesicles at the start of the reaction. This would effectively eliminate inhibition of SERCA caused by Ca^2+^ accumulated in the vesicles prior to the start of the reaction. Further, the initial rate of SERCA-mediated Ca^2+^ uptake, before product inhibition occurs, is inhibited. Lastly, our team discovered in an animal model of SE that SERCA-mediated Ca^2+^ uptake was inhibited immediately following SE as was in the present cell culture study and also remained inhibited 1 year later during the chronic phase of epilepsy [20,21].

Since homogenate proteins are balanced [31] prior to Ca^2+^ uptake assays and the protein balancing procedure does not distinguish live and dead neurons, it was important to evaluate neuronal viability in hippocampal cultures after low Mg^2+^ treatment. Neurons were blindly evaluated immediately after 3 h of low Mg^2+^ treatment and prior to homogenization utilizing the criteria of phase brightness [18,25,26]. Employing this method offers the advantage of evaluating cell viability and ER vesicle Ca^2+^ uptake capacity within the same culture dish. It was found that there was no significant variation in neuronal viability between cultures treated with control or low Mg^2+^ solutions, consistent with previous results [18,26]. Thus, inhibition of SERCA-mediated Ca^2+^ uptake into ER vesicles was not an artifact of decreased neuronal viability in hippocampal cultures subjected to low Mg^2+^ treatment.

Primary cultures used in the present study consisted of hippocampal neurons grown on a confluent bed of hippocampal glia. The amount of glia plated contributed approximately 16% of the total amount of tissue in the culture dish. Thus, it was important to determine the effect of 3 h of low Mg^2+^ treatment on glial cells and the contribution of this effect on inhibition of Ca^2+^ uptake observed in N+G culture homogenates. Significant inhibition of both the total capacity and initial rate of Ca^2+^ uptake was observed in homogenates from glial cultures treated with low Mg^2+^ compared to control (Figure 4). However, comparison of the total Ca^2+^ uptake capacities of N+G (Figure 2) to glial (Figure 4) homogenates revealed that glial ER vesicles contributed only 20% of the Ca^2+^ uptake activity observed in the N+G homogenates, consistent with the percentage of total tissue in N+G dishes that is glial in origin. These data show that ER SERCA-mediated Ca^2+^ sequestration and low Mg^2+^-induced inhibition observed in N+G homogenates is predominately neuronal in origin.

Removal of the Mg^2+^ antagonist during exposure of hippocampal neurons to low Mg^2+^ media leads to prolonged and excessive activation of the NMDA receptor. It has been demonstrated that multifunctional Ca^2+^/calmodulin-dependent protein kinase II (CaM kinase II) phosphorylates and activates SERCA in cardiac tissue [38]. It was also shown that the same isoform of SERCA found in cardiac tissue is also found in brain [39]. Additionally, it was observed that CaM kinase II expression and activity is decreased in models of SE and epilepsy [40,41,42] and that inhibition of Cam kinase II is a result of excitotoxic activation of the NMDA receptor [43]. It is, therefore, feasible that extended NMDA receptor hyperactivity that leads to decreased expression/activity of Cam kinase II results in decreased phosphorylation of SERCA and inhibition of ER Ca^2+^ sequestration.

There are a multitude of conceivable repercussions of inhibition of ER SERCA-mediated Ca^2+^ sequestration. Calcineurin is a Ca^2+^-stimulated phosphatase that is a critical player in the delicate balance of phosphorylation/dephosphorylation regulated signal transduction and plasticity in hippocampal neurons [44]. It was shown in the rat pilocarpine model that hippocampal calcineurin activity was significantly elevated and translocated to the plasma membrane fraction after prolonged SE [45,46]. It was also demonstrated that [Ca^2+^]_i_ became sustained and elevated in hippocampal cells as a result of prolonged seizures and that inhibition of ER SERCA Ca^2+^ uptake was involved [15,30]. Thus, it is conceivable that inhibition of ER SERCA activity would have detrimental consequences to the activity of calcineurin. The ER is also a critical step in protein trafficking [47] and unchecked deregulation of ER Ca^2+^ homeostasis leads to the unfolded protein response [48]. It was demonstrated that both excitatory [49] and inhibitory [50] neurotransmitter receptors are dependent on specific ER signals for correct transport to the plasma membrane. It was also shown in the same model utilized in this study that gamma-aminobutyric acid type A receptor expression was significantly decreased as a result of prolonged exposure to low Mg^2+^ solution [13]. Therefore, it is plausible that disruption of normal ER functioning through inhibition of SERCA activity could significantly affect the transport of important neurotransmitter receptors to the plasma membrane surface. The myriad of possible downstream effects caused by ER deregulation warrant research into the molecular mechanisms of SERCA inhibition induced by SE.

## 5. Conclusions

Utilizing a well established cell culture model of status epilepticus in hippocampal neurons, it was shown that SERCA mediated Ca^2+^ sequestration into ER vesicles was significantly inhibited in association with a 3 h exposure to low Mg^2+^ conditions. Viability of neurons following incubation in both control and low Mg^2+^ solutions for 3 h was not different. Both the initial rate and steady-state activity of SERCA were inhibited. The glial bed that the neurons were grown on also showed inhibition of Ca^2+^ uptake, but the inhibition observed in the mixed neuronal/glial cultures was mostly neuronal in origin. The degree of SERCA inhibition was concomitant with duration of low Mg^2+^ exposure as was the level of neuronal cytosolic Ca^2+^, as Mg^2+^ exposure increased from 1 h to 3 h, inhibition of SERCA and levels of cytosolic Ca^2+^ likewise increased.

## References

[1] Gurcharran K., Grinspan Z.M. (2019). The burden of pediatric status epilepticus: Epidemiology, morbidity, mortality, and costs. Seizure.

[2] Nelson S.E., Varelas P.N. (2018). Status Epilepticus, refractory status epilepticus, and super-refractory status epilepticus. Contin. Lifelong Learn. Neurol..

[3] Mele M., Ribeiro L., Inacio A.R., Wieloch T., Duarte C.B. (2014). GABA(A) receptor dephosphorylation followed by internalization is coupled to neuronal death in in vitro ischemia. Neurobiol. Dis..

[4] Kumar A. (2020). Calcium signaling during brain aging and its influence on the hippocampal synaptic plasticity. Adv. Exp. Med. Biol..

[5] Huang Y.Z., He X.P., Krishnamurthy K., McNamara J.O. (2019). TrkB-Shc signaling protects against hippocampal injury following status epilepticus. J. Neurosci..

[6] Griffiths T., Evans M.C., Meldrum B.S. (1982). Intracellular sites of early calcium accumulation in the rat hippocampus during status epilepticus. Neurosci. Lett..

[7] Deshpande L.S., DeLorenzo R.J. (2020). Novel therapeutics for treating organophosphate-induced status epilepticus co-morbidities, based on changes in calcium homeostasis. Neurobiol. Dis..

[8] Choi H.S., Lee C.H. (2016). Time-course changes of hippocalcin expression in the mouse hippocampus following pilocarpine-induced status epilepticus. J. Vet. Sci..

[9] Delorenzo R.J., Sun D.A., Deshpande L.S. (2005). Cellular mechanisms underlying acquired epilepsy: The calcium hypothesis of the induction and maintainance of epilepsy. Pharmacol. Ther..

[10] Phillips K.F., Deshpande L.S., DeLorenzo R.J. (2018). Hypothermia reduces mortality, prevents the calcium plateau, and is neuroprotective following status epilepticus in rats. Front. Neurol..

[11] Xiang L., Ren Y., Cai H., Zhao W., Song Y. (2015). MicroRNA-132 aggravates epileptiform discharges via suppression of BDNF/TrkB signaling in cultured hippocampal neurons. Brain Res..

[12] Sombati S., Delorenzo R.J. (1995). Recurrent spontaneous seizure activity in hippocampal neuronal networks in culture. J. Neurophysiol..

[13] Blair R.E., Sombati S., Lawrence D.C., McCay B.D., DeLorenzo R.J. (2004). Epileptogenesis causes acute and chronic increases in GABAA receptor endocytosis that contributes to the induction and maintenance of seizures in the hippocampal culture model of acquired epilepsy. J. Pharmacol. Exp. Ther..

[14] DeLorenzo R.J., Pal S., Sombati S. (1998). Prolonged activation of the N-methyl-D-aspartate receptor-Ca^2+^ transduction pathway causes spontaneous recurrent epileptiform discharges in hippocampal neurons in culture. Proc. Natl. Acad. Sci. USA.

[15] Pal S., Sombati S., Limbrick D.D., DeLorenzo R.J. (1999). In vitro status epilepticus causes sustained elevation of intracellular calcium levels in hippocampal neurons. Brain Res..

[16] Handforth A., Treiman D.M. (1995). Functional mapping of the early stages of status epilepticus: A 14C-2-deoxyglucose study in the lithium-pilocarpine model in rat. Neuroscience.

[17] Smith Z.Z., Benison A.M., Bercum F.M., Dudek F.E., Barth D.S. (2018). Progression of convulsive and nonconvulsive seizures during epileptogenesis after pilocarpine-induced status epilepticus. J. Neurophysiol..

[18] Blair R.E., Churn S.B., Sombati S., Lou J.K., DeLorenzo R.J. (1999). Long-lasting decrease in neuronal Ca^2+^/calmodulin-dependent protein kinase II activity in a hippocampal neuronal culture model of spontaneous recurrent seizures. Brain Res..

[19] Pedersen P.L., Carafoli E. (1987). Ion motive ATPases. I. Ubiquity, properties, and significance to cell function. Trends Biochem. Sci..

[20] Parsons J.T., Churn S.B., DeLorenzo R.J. (2001). Chronic inhibition of cortex microsomal Mg(2+)/Ca(2+) ATPase-mediated Ca(2+) uptake in the rat pilocarpine model following epileptogenesis. J. Neurochem..

[21] Parsons J.T., Churn S.B., Kochan L.D., DeLorenzo R.J. (2000). Pilocarpine-induced status epilepticus causes N-methyl-D-aspartate receptor-dependent inhibition of microsomal Mg(2+)/Ca(2+) ATPase-mediated Ca(2+) uptake. J. Neurochem..

[22] Parsons J.T., Churn S.B., DeLorenzo R.J. (1997). Ischemia-induced inhibition of calcium uptake into rat brain microsomes mediated by Mg^2+^/Ca^2+^ ATPase. J. Neurochem..

[23] Parsons J.T., Sun D.A., DeLorenzo R.J., Churn S.B. (2004). Neuronal-specific endoplasmic reticulum Mg(2+)/Ca(2+) ATPase Ca(2+) sequestration in mixed primary hippocampal culture homogenates. Anal. Biochem..

[24] Sun D.A., Sombati S., Blair R.E., DeLorenzo R.J. (2002). Calcium-dependent epileptogenesis in an in vitro model of stroke-induced “epilepsy”. Epilepsia.

[25] Limbrick D.D., Churn S.B., Sombati S., DeLorenzo R.J. (1995). Inability to restore resting intracellular calcium levels as an early indicator of delayed neuronal cell death. Brain Res..

[26] Gibbs J.W., Sombati S., DeLorenzo R.J., Coulter D.A. (1997). Physiological and pharmacological alterations in postsynaptic GABA(A) receptor function in a hippocampal culture model of chronic spontaneous seizures. J. Neurophysiol..

[27] Coulter D.A., Sombati S., DeLorenzo R.J. (1992). Electrophysiology of glutamate neurotoxicity in vitro: Induction of a calcium-dependent extended neuronal depolarization. J. Neurophysiol..

[28] Hamill O.P., Marty A., Neher E., Sakmann B., Sigworth F.J. (1981). Improved patch-clamp techniques for high-resolution current recording from cells and cell-free membrane patches. Pflug. Arch..

[29] Limbrick D.D., Pal S., DeLorenzo R.J. (2001). Hippocampal neurons exhibit both persistent Ca2+ influx and impairment of Ca^2+^ sequestration/extrusion mechanisms following excitotoxic glutamate exposure. Brain Res..

[30] Pal S., Sun D., Limbrick D., Rafiq A., DeLorenzo R.J. (2001). Epileptogenesis induces long-term alterations in intracellular calcium release and sequestration mechanisms in the hippocampal neuronal culture model of epilepsy. Cell Calcium.

[31] Bradford M.M. (1976). A rapid and sensitive method for the quantitation of microgram quantities of protein utilizing the principle of protein-dye binding. Anal. Biochem..

[32] Morris G., Leite M., Kullmann D.M., Pavlov I., Schorge S., Lignani G. (2017). Activity clamp provides insights into paradoxical effects of the anti-seizure drug carbamazepine. J. Neurosci..

[33] Deshpande L.S., DeLorenzo R.J. (2011). Acetaminophen inhibits status epilepticus in cultured hippocampal neurons. Neuroreport.

[34] Parsons J.T., Churn S.B., DeLorenzo R.J. (1999). Global ischemia-induced inhibition of the coupling ratio of calcium uptake and ATP hydrolysis by rat whole brain microsomal Mg(2+)/Ca(2+) ATPase. Brain Res..

[35] Miller R.J. (1991). The control of neuronal Ca^2+^ homeostasis. Prog. Neurobiol..

[36] Thastrup O., Cullen P.J., Drobak B.K., Hanley M.R., Dawson A.P. (1990). Thapsigargin, a tumor promoter, discharges intracellular Ca^2+^ stores by specific inhibition of the endoplasmic reticulum Ca2(+)-ATPase. Proc. Natl. Acad. Sci. USA.

[37] Trotta E.E., de Meis L. (1975). ATP-dependent calcium accumulation in brain microsomes. Enhancement by phosphate and oxalate. Biochim. Biophys. Acta.

[38] Xu A., Hawkins C., Narayanan N. (1993). Phosphorylation and activation of the Ca(2+)-pumping ATPase of cardiac sarcoplasmic reticulum by Ca^2+^/calmodulin-dependent protein kinase. J. Biol. Chem..

[39] Lytton J., Westlin M., Burk S.E., Shull G.E., MacLennan D.H. (1992). Functional comparisons between isoforms of the sarcoplasmic or endoplasmic reticulum family of calcium pumps. J. Biol. Chem..

[40] Bronstein J.M., Farber D.B., Wasterlain C.G. (1993). Regulation of type-II calmodulin kinase: Functional implications. Brain Res. Rev..

[41] Murray K.D., Gall C.M., Benson D.L., Jones E.G., Isackson P.J. (1995). Decreased expression of the alpha subunit of Ca^2+^/ calmodulin-dependent protein kinase type II mRNA in the adult rat CNS following recurrent limbic seizures. Brain Res. Mol. Brain Res..

[42] Xu Y., Li Z., Yao L., Zhang X., Gan D., Jiang M., Wang N., Chen G., Wang X. (2017). Altered norbin expression in patients with epilepsy and a rat model. Sci. Rep..

[43] Churn S.B., Limbrick D., Sombati S., DeLorenzo R.J. (1995). Excitotoxic activation of the NMDA receptor results in inhibition of calcium/calmodulin kinase II activity in cultured hippocampal neurons. J. Neurosci..

[44] Winder D.G., Sweatt J.D. (2001). Roles of serine/threonine phosphatases in hippocampal synaptic plasticity. Nat. Rev. Neurosci..

[45] Kurz J.E., Sheets D., Parsons J.T., Rana A., Delorenzo R.J., Churn S.B. (2001). A significant increase in both basal and maximal calcineurin activity in the rat pilocarpine model of status epilepticus. J. Neurochem..

[46] Kurz J.E., Rana A., Parsons J.T., Churn S.B. (2003). Status epilepticus-induced changes in the subcellular distribution and activity of calcineurin in rat forebrain. Neurobiol. Dis..

[47] Wang B., Stanford K.R., Kundu M. (2020). ER-to-golgi trafficking and its implication in neurological diseases. Cells.

[48] Saito A., Imaizumi K. (2018). The broad spectrum of signaling pathways regulated by unfolded protein response in neuronal homeostasis. Neurochem. Int..

[49] Ren Z., Riley N.J., Needleman L.A., Sanders J.M., Swanson G.T., Marshall J. (2003). Cell surface expression of GluR5 kainate receptors is regulated by an endoplasmic reticulum retention signal. J. Biol. Chem..

[50] Kang J.Q., Macdonald R.L. (2004). The GABAA receptor gamma2 subunit R43Q mutation linked to childhood absence epilepsy and febrile seizures causes retention of alpha1beta2gamma2S receptors in the endoplasmic reticulum. J. Neurosci..

